# Metastasizing Pleomorphic Adenoma: A Rare Entity

**DOI:** 10.7759/cureus.78085

**Published:** 2025-01-27

**Authors:** Amar Hazwan Zainal Ariffin, Sai Guan Lum, Ng Chong Sian, Soon Ching Gan

**Affiliations:** 1 Department of Otorhinolaryngology - Head and Neck Surgery, Universiti Kebangsaan Malaysia Medical Centre, Kuala Lumpur, MYS; 2 Faculty of Medicine, National University of Malaysia, Kuala Lumpur, MYS; 3 Department of Otorhinolaryngology, Hospital Tengku Ampuan Rahimah, Klang, MYS; 4 Department of Pathology, Hospital Tengku Ampuan Rahimah, Klang, MYS

**Keywords:** case report, metastasizing pleomorphic adenoma, parotid gland, pleomorphic adenoma, salivary gland

## Abstract

Metastatic pleomorphic adenoma (MPA) is rare and one of the outcomes of long-standing pleomorphic adenoma (PA). Due to its rarity, its prevalence is not well understood.

While PA usually presents in the second decade of life, earlier onset may increase the risk of MPA. In cases of recurrent PA, a high suspicion for MPA is advised, and full-body imaging is recommended to detect metastasis. Enucleation is not recommended due to its association with higher recurrence rates, and total surgical excision, such as parotidectomy, is preferred. Chemotherapy and radiation are ineffective for MPA, and surgical excision is the primary treatment. Despite surgical intervention, recurrence and metastasis can still occur, with a reported 50% mortality rate at five years and a World Health Organization (WHO) report indicating that 40% of patients die from the disease.

Hereby, we report a patient who presented with a recurrent parotid gland PA that metastasized to the ipsilateral lymph node nine years after the initial treatment.

## Introduction

Salivary gland tumors are rare, comprising only 1%-4% of all neoplasms [1]. Among these, pleomorphic adenoma (PA) is the most common neoplasm that affects the salivary glands [2]. Long-standing PA can transform into three forms of malignancy, including metastasizing pleomorphic adenoma (MPA), carcinosarcoma, and carcinoma ex-pleomorphic adenoma (CEPA) [3].

According to the World Health Organization (WHO), MPA is described as a "histologically benign pleomorphic adenoma that mysteriously displays local or distant metastasis" [4]. This is bizarre for a benign neoplasm such as a pleomorphic adenoma but capable of spreading to other parts of the body, which is a feature of malignant neoplasm [2].

Despite appearing benign in histology, MPA behaves aggressively, with a mortality rate as high as 22% [5]. The majority of MPA cases occur in the parotid gland (74%), followed by minor salivary glands (17%) and submandibular glands (10%) [2]. Metastasis occurs most frequently in bones (45%), followed by the head and neck (43%), lungs (36%), and abdominal viscera (10%) [2]. Only 17% of MPA metastasizes to regional lymph nodes [2].

However, distant metastasis of MPA is not commonly recognized and is frequently missed. At least one local recurrence of PA occurred prior to the discovery of distant metastasis in 81% of MPA patients, and only 17% of patients were diagnosed with MPA during a medical assessment of a recurring PA [2].

Although PA is a relatively benign tumor, its long-standing presence or recurrence can lead to MPA, characterized by the dissemination of otherwise histologically benign tumor cells to distant or regional sites. Due to its rarity, the epidemiology and risk factors of MPA remain poorly understood, adding to the challenges in its clinical management.

## Case presentation

A 28-year-old female patient of Kadazan ethnicity presented to the otorhinolaryngology (ORL) clinic at Hospital Tengku Ampuan Rahimah with multiple painless right neck swellings that gradually increased for one year. There were no other concurrent symptoms such as discharge, facial weakness, or weight loss. The patient had a history of right superficial parotidectomy for a pleomorphic adenoma in 2013.

Clinical examination revealed a lobulated right neck mass at the infra-auricular and angle of the mandible region, which measured 2 × 3 cm. The mass was firm and not tender with a regular and smooth surface but appeared fixed to the underlying structure.

The fine needle aspiration cytology smear of the mass showed predominant plasmacytoid myoepithelial cells and spindle myoepithelial cells with a magenta fibrillary matrix. The cells showed slightly enlarged nuclei with small, prominent nucleoli and mild variation in size. The cytoplasm was moderate in amount. Epithelial cells with sheets, tubules, and acini formation were noted. No mitotic figures were seen. The findings were suggestive of salivary gland neoplasm of uncertain malignant potential.

The initial working diagnosis was right parotid CEPA, and thus, a contrast-enhanced computed tomography (CECT) scan was performed.

**Figure 1:**
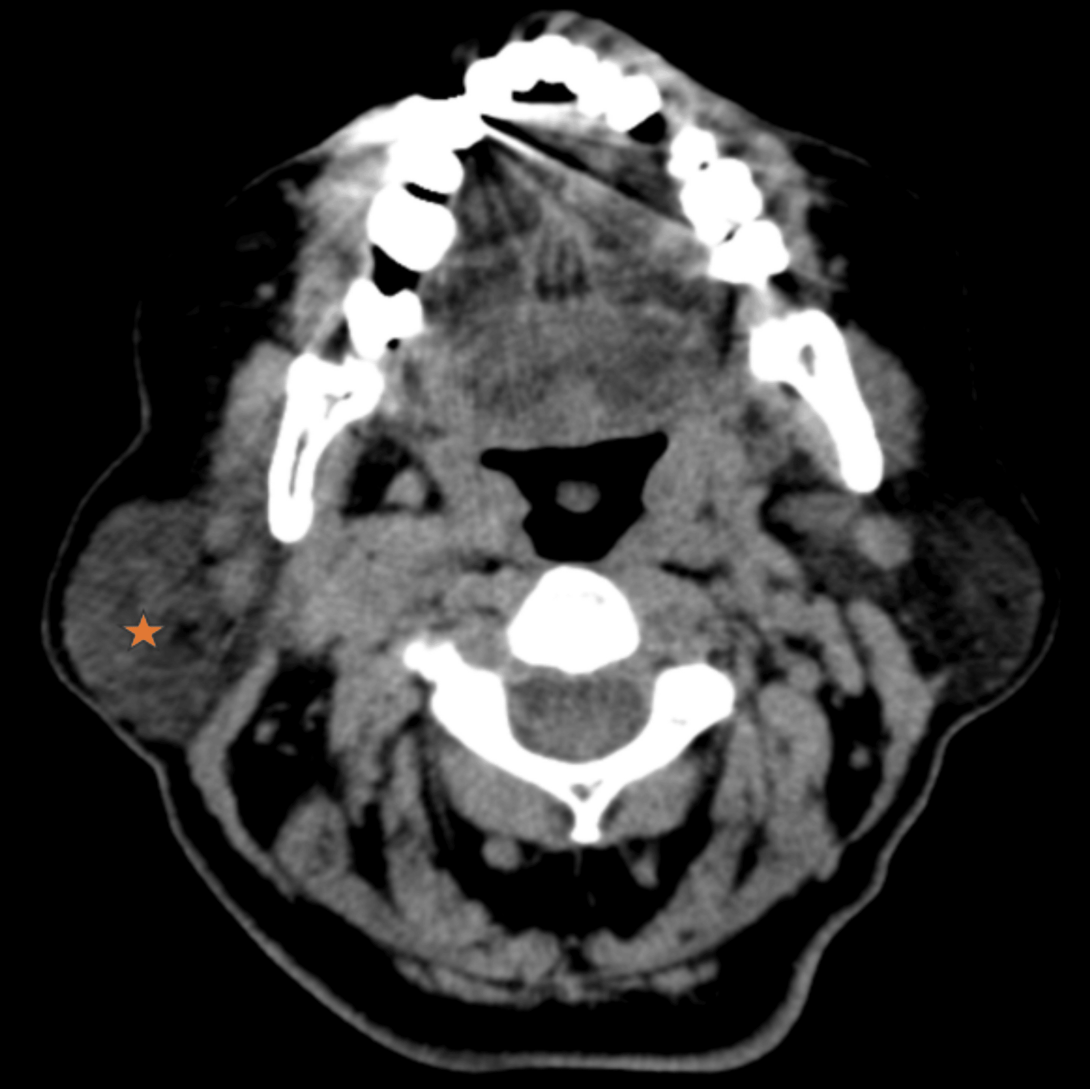
Contrast-enhanced CT of the neck showing right lobulated parotid mass (star). CT: computed tomography

The CECT revealed a multilobulated heterogeneously enhancing uncalcified mass at the right parotid gland, measuring 4.2 × 2 × 4.4 cm (Figure 1). This tumor extended superiorly to the level of the right temporomandibular joint; however, there was no bone erosion or intracranial extension. There was no medial extension to the deep lobe of the parotid gland. The mass extended inferiorly until the C3/C4 level with multiple enlarged cervical nodes on the right Ia, Ib, and II. Cervical node IIa was the largest on the right (inferior to the mass), measuring 2 × 3 cm. No evidence of distance metastasis was found.

**Figure 2:**
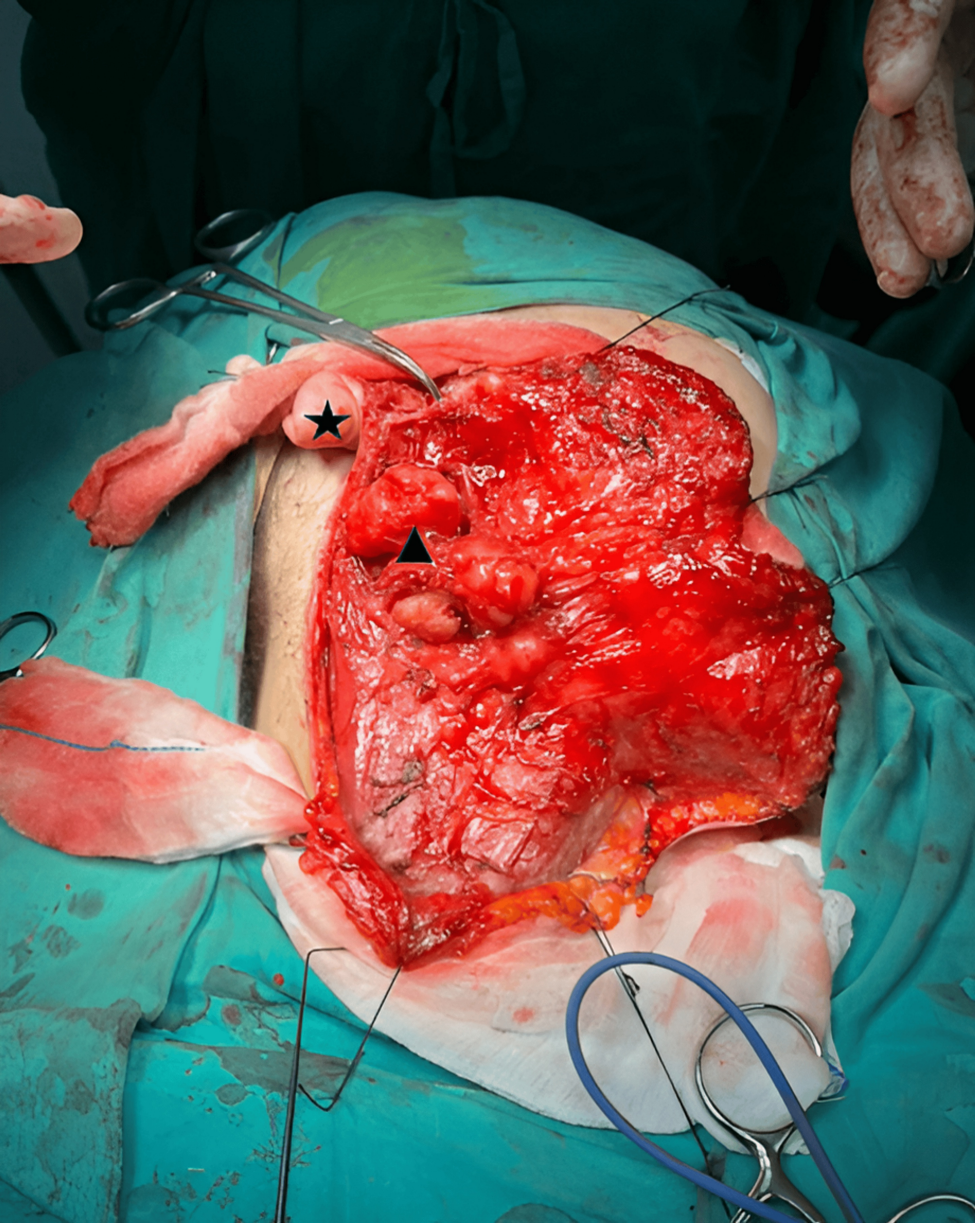
Intraoperative image of the right lobulated parotid mass (triangle) below the right ear lobule (star).

Total parotidectomy and right selective neck dissection were performed. Intraoperatively, there was a lobulated right parotid tumor arising from the deep lobe, and the main trunk of the facial nerve could not be identified in view of distorted anatomy. However, the cervicofacial, marginal mandibular, and buccal nerves were identified and preserved (Figure 2). Despite intraoperative identification and preservation of the marginal mandibular nerve, postoperatively, the patient developed right marginal mandibular nerve palsy.

On macroscopic examination of the tumor, there were three lobulated firm masses measuring 50 × 35 × 15 mm, 25 × 15 × 12 mm, and 6 × 5 × 5 mm, with no attached fibrofatty tissue or normal parotid gland tissue seen. On serial sectioning, the masses showed a solid whitish to cream-tan cut surface with occasional myxoid areas. No necrosis was seen.

**Figure 3:**
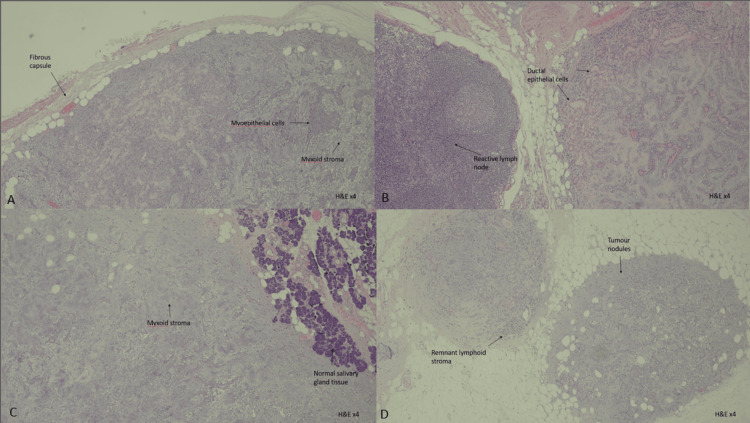
A: Pleomorphic adenoma encircled by fibrous capsule. B: Pleomorphic adenoma nodule with adjacent lymph node. C: Pleomorphic adenoma with adjacent normal salivary gland. D: Pleomorphic adenoma to adjacent lymph node.

Microscopic examination revealed an encapsulated triphasic tumor composed of epithelial cells, myoepithelial cells, and stromal components in a mixture of patterns (Figure 3). The spindled myoepithelial cells streamed from the ductal elements into the chondromyxoid stroma. The myoepithelial cells showed a spectrum of phenotypes, including oval, spindled, epithelioid, and plasmacytoid. Squamous metaplasia and scattered calcifications were also noted. No ductal atypia, diffuse fibrosis, increased mitosis, necrosis, high-grade component, or malignancy were seen. The tumor cells were present focally at the inked excision margins.

Out of 33 lymph nodes identified, six lymph nodes from levels IIa and III showed salivary gland parenchyma with numerous tumor nodules of varying sizes composed of neoplastic cells, as described earlier. No tumor metastasis was seen in other samples taken.

Thus, the impression of metastasizing pleomorphic adenoma (MPA) was made based on the histopathology report, and the patient was referred to an oncologist for further management. However, as the primary treatment is surgical removal, no chemotherapy or radiotherapy was offered to the patient.

During surveillance follow-ups every six months, the patient did not complain of symptom recurrence or any new complaint. The postoperative wound was well healed, and there was slight marginal mandibular nerve palsy, which improved.

## Discussion

The most widespread benign salivary gland lesions are pleomorphic adenomas. They grow slowly and do not typically spread to other regions of the body. In some instances, however, pleomorphic adenomas can recur or become malignant and spread to other areas of the body, resulting in metastatic pleomorphic adenoma. Rarely, particularly when insufficient excision is performed, can pleomorphic adenomas metastasize without undergoing a malignant histological transformation [2].

Some aspects of this disorder are still the subject of debate, and its prevalence is unknown. Knight and Ratnasingham performed a systemic review and discovered that there were 80 known MPA cases worldwide from 1942 to 2014, with an average age of diagnosis at 49.5 years [6]. McGarry et al. analyzed patients with MPA for a period of 50 years and observed only 52 cases until 2007 [7]. Livolsi and Perzin examined 47 individuals with an average age of 59 (range: 34-86) years and discovered that there were more female patients than male patients (35 versus 12) [8].

According to Knight and Ratnasingham, the average age of PA presentation in patients with MPA is 34.3 years, with a range of 9-73 years, and the most common for primary PA is the second decade [6]. In this instance, the patient was 19 when she was diagnosed with PA.

Local recurrence of PA was reported before MPA in 72.8% of cases, with 37% demonstrating multiple local recurrences [6]. Between the presentation of the initial PA and the identification of the recurrence or distant metastases, Knight and Ratnasingham [6] and McGarry et al. [7] experienced latency periods of 16 and 14.9 years, respectively. As in the present case, the local recurrence occurred nine years after the primary PA.

In cases of persistent or recurrent pleomorphic adenomas, a high index of suspicion for MPA is advised, and additional tests, including full-body CT scans, magnetic resonance imaging, and/or positron emission tomography (PET) scans, should be performed to rule out metastasis [7]. In this instance, only a neck CECT was performed, as the initial suspicion was for CEPA, and the patient had no other systemic complaints.

Enucleation should not be the treatment of choice for PA, as inadequate tumor clearance is strongly associated with local recurrence and tumor cell leakage increases the risk of MPA [2]. Reiland et al. endorse entire surgical excision, such as parotidectomy [9]. After enucleation, the reported local recurrence rate is 20%-45%, whereas it is 1%-5% after total parotidectomy [10]. Despite undergoing surgical excision of PA, the patient developed recurrence and metastasis to the ipsilateral cervical neck node.

The vast majority of patients with MPA underwent total surgical removal, while only a tiny percentage received nonoperative care. A log-rank analysis revealed that surgical treatment enhanced survival significantly over nonoperative treatment [2]. Chemotherapy and primary radiation were ineffective against this disease [2]. This is also supported by the systemic review by Knight and Ratnasingham, which concludes that there is no solid evidence that combining radiation and surgery would enhance survival rates [6]. Thus, the patient only received surgical excision without receiving chemotherapy or radiotherapy.

At five years, mortality reaches 50% [7]. WHO reports that 40% of patients perish from the disease, 47% live disease-free, and 13% live with the disease [4].

## Conclusions

Although pleomorphic adenoma is a benign salivary gland tumor, metastases can occur years later in conjunction with local recurrences. There are no histological markers that can predict MPA. However, local recurrence following surgical enucleation of pleomorphic adenoma has been linked to an increased risk of developing MPA. A complete excision of pleomorphic adenoma is warranted to reduce the possibility of distant dissemination. In cases of persistent or recurring pleomorphic adenomas, a high index of suspicion for MPA is suggested, and additional tests such as full-body CT scans, magnetic resonance imaging, and/or PET scans should be performed to rule out metastasis.
